# Need for Recovery and Work–Family Conflict in the Armed Forces: A Latent Profile Analysis of Job Demands and Resources

**DOI:** 10.3390/ijerph22050795

**Published:** 2025-05-18

**Authors:** Francesco Pace, Cristina Moavero, Giuditta Cusimano, Giulia Sciotto

**Affiliations:** 1 Department of Economics, Business and Statistics, University of Palermo, Viale delle Scienze ed. 13, 90128 Palermo, Italy; giulia.sciotto@unipa.it; 2 Department of Physics and Chemistry, University of Palermo, Viale delle Scienze ed. 17, 90128 Palermo, Italy; cristina.moavero@unipa.it (C.M.); giuditta.cusimano@unipa.it (G.C.)

**Keywords:** job demands–resources model, armed forces, need for recovery, work–family conflict, work-related stress, well-being

## Abstract

Building on the Job Demands–Resources model, this study aims to investigate the interaction between job characteristics and work-related stress indicators in a sample of 211 Italian Armed Forces personnel. Using Latent Profile Analysis (LPA), three distinct profiles emerged based on job demands (workload and perception of performing high-risk work) and resources (relationships with colleagues and supervisors, perceived meaningfulness of work, and feedback received on quality of work). The first profile, characterized by high demands and low resources, was associated with higher levels of work–family conflict and need for recovery (an indicator of perceived fatigue at the end of the workday). The second profile, characterized by high resources and low demands, showed the lowest levels of both need for recovery and work–family conflict, while the third profile showed average scores on demands, resources, and outcome variables. These findings are in line with the chosen theoretical framework and prompt several reflections on work-related well-being in the armed forces and what can promote it. Stemming from the results is the importance of organizational interventions designed to protect employees’ well-being and support their effective functioning. Such interventions are particularly critical within the armed forces context, where operational performance has a direct impact on the safety and well-being of citizens.

## 1. Introduction

The literature on work-related stress in the armed forces and law enforcement agencies has revealed numerous factors that influence the delicate balance between work and personal life, in addition to all the unique challenges and pressures that characterize military service and can hardly be changed. Specifically, the workload and risks associated with armed forces and police duties are associated with higher levels of psychological and physical distress, and yet they cannot be avoided as defining elements of the military work environment [1,2]. The need in these cases is to manage and modulate them through variables that can act as a resource, support, or buffer. Research in this field shows that both individual and organizational characteristics play a crucial role in predicting the possibility of maintaining a satisfactory work–life balance [3,4]. A central aspect in this regard is the importance of organizational support and interpersonal relationships that are established within the work context, and which in the military gain particular significance [5]. The issue of work-related fatigue is a pressing concern for armed forces personnel. Fatigue is intrinsically linked to work–life balance because, if the fatigue experienced during the workday is not adequately recovered, it will negatively affect the personal sphere, threatening the ability to detach from work-related thoughts even during leisure time [6]. Finding a balance seems to be particularly difficult for armed forces personnel, who may find the transition from a highly structured military to civilian life context particularly difficult [7]. The strong interconnection between work and family life underscores the need for policies that support military personnel in a variety of contexts. Adequate recognition and support of mental health issues not only improve overall quality of life but also influence job performance [8,9].

This study aims to analyze the role of some job demand and resource variables in work–life conflict and the need for energy recovery at the end of the working day among personnel of the Italian Armed Forces and law enforcement agencies. The starting hypothesis, based on what the Job Demands–Resources model [10] postulates, is that an imbalance between demands and resources leads to negative outcomes, such as work–family conflict and increased need to recover energy at the end of the workday. The Job Demands–Resources (JD-R) model [10] provides an insightful lens through which to analyze the relationship between job resources (such as social support from colleagues and supervisors, meaningful work, and feedback) and job demands (such as workload and perception of performing a risky job). Various studies underscore the fundamental role these job resources play in mitigating the potential negative impacts of job demands—especially within the unique context of military service. The JD-R framework emphasizes the importance of job resources in countering the effects of job demands, which directly impact employees’ psychological health and their ability to effectively manage both work and personal life [11]. The impact of job demands is particularly detrimental in high-pressure work environments, where employees face not only the stress of their immediate job responsibilities but also perceptions of being involved in high-risk settings [12,13,14]. Job demands, if not adequately managed, influence employees’ well-being, performance, and overall experience in the workplace [11]. As a result, there is a need to assess both the demands and the resources available to workers in order to manage the consequences in terms of physical and mental health, as well as to guide the direction of management and prevention measures.

### 1.1. The Role of Workload and the Perception of Work-Related Risks

Research has identified consistent relationships between high job demands and negative outcomes, including increased work–life conflict and a greater need for recovery [12,13,14,15]. Having to sustain high workloads is commonly cited as a stressor directly related to negative outcomes such as stress and fatigue, which inevitably deplete physical and psychological resources over time and lead to worsening states [16]. This is the process of health deterioration theorized by the JD-R model [11]. Excessive workloads contribute to significant difficulties in balancing work and personal life, further exacerbating the likelihood of burnout and stress [12,16]. This is in line with the findings of studies suggesting that workers dealing with high demands need more psychological recovery time to mitigate these negative experiences [15,17]. Often, the need for psychological recovery affects the quality of leisure time available to workers and is associated with problems in their family life [18,19]. In particular, the specificity of the military environment often requires workers in the armed forces to prioritize their duties over personal and family life, contributing significantly to conflicts between work and domestic responsibilities [20].

A recent study highlights that excessive job demands directly contribute to emotional exhaustion, reinforcing the idea that military personnel require substantial recovery periods after demanding roles [21]. This may be further exacerbated by prolonged deployments, which have been shown to lead to adverse psychological outcomes [22]. Deployment conditions create psychological pressures that necessitate effective recovery strategies to deal with fatigue and stress-related disorders [23]. Military personnel may hesitate to seek mental health support due to fears about the implications for their careers [2]. This hesitance can contribute to amplifying the psychological burden they have to bear [2]. Consequently, the lack of sufficient recovery options presents significant challenges in addressing fatigue and work-related stress, as well as in attempting to balance work and private spheres.

Moreover, professions with inherent risks, such as the armed forces or emergency services, often feature a work environment in which the pressure to maintain consistent levels of performance is very strong [12,13,14]. In a study that examined police work, emotional and psychological demands related to the perception of danger were found to be directly related to increased levels of work-related stress and subsequent work–family conflict [24]. When workers perceive their work as dangerous, they may experience high anxiety and constant vigilance, resulting in a higher need for recovery after work hours. This continuous state of agitation can disrupt family life and obligations, leading to further conflict between work and domestic responsibilities [25,26]. Workers in the armed forces are regularly exposed to potentially dangerous situations, which amplifies the psychological burden of their work compared to other occupations [27,28]. Numerous studies have pointed out that the inherent unpredictability of high-risk occupations activates chronic physiological responses that contribute to stress accumulation [28,29]. This dynamic is complicated by subjective risk assessment. For example, some soldiers may develop high-risk tolerance through cumulative exposure and training [27]. Conversely, other individuals may be more sensitive to perceived risk, resulting in greater psychological and physiological reactions to stress [30]. Research in the armed forces has suggested that such individual variability is critical in determining not only short-term stress responses but also long-term outcomes in terms of well-being and job satisfaction [30]. High-risk occupations are often characterized more by the anticipation of potential danger than by the risk itself, resulting in prolonged activation of the stress response system [27]. Chronic stress induced by the perception or anticipation of danger has been shown to erode psychological resilience over time, making long-term recovery particularly difficult [28,29]. The effects of occupational risk perception extend beyond the work sphere and spill over into workers’ personal lives, often contributing to strained interpersonal relationships and reduced overall life satisfaction [31,32]. When the psychological burden of perceived danger is not adequately managed, it often intrudes into family life and social interactions [32]. This spillover can lead to family conflict, erode social support networks, and further contribute to the vicious cycle of job stress [31]. According to the JD-R model [10], the expectation of risk itself constitutes a “demand” that requires emotional, cognitive, or physical resources to be managed [31]. Researchers have pointed out that when the balance between job demands and available resources (or received support) decays, the resulting imbalance may cause a range of deleterious health outcomes [31]. This model [10] thus provides a theoretical basis for understanding the unique stress profiles observed in high-risk military occupations.

### 1.2. The Role of Social Relationships, Feedback, and Meaningful Work

There is vast empirical evidence suggesting that an imbalance between job demands and available resources can have negative effects on workers’ well-being [11]. When demands are high and resources are scarce, stress levels tend to rise, and the conflict between work and personal life increases [33]. However, the implementation of effective feedback mechanisms, supportive relationships, and an organizational culture that fosters meaningful work can help to create a more sustainable work environment, improve employees’ well-being, and promote work–life balance [19,34].

In the military context, these dynamics are even more critical. Military personnel often face high workloads and extended hours, which can strain personal well-being and family relationships [35]. On the other hand, the availability of adequate resources plays a crucial role in enabling workers to better manage professional challenges [11]. Greater work resources are also associated with less interference between work and personal life during periods of high work demands [36]. For example, the presence of social support (e.g., solid relationships with supervisors and colleagues) can alleviate the negative effects of high demands through a buffering effect [37,38]. Receiving support from colleagues enables service members to manage stress more effectively, allowing them to recover adequately during downtime and maintain healthier relationships with their families [39,40]. In the military context, social support from colleagues and supervisors is a fundamental resource, as living in unpredictable environments requires adequate tools to manage stress and strengthen individual resilience [4]. For example, soldiers who perceive their supervisors as supportive tend to report lower levels of work-related stress and, consequently, experience fewer conflicts between professional and family obligations [41]. Similarly, another study observed how informal support networks among military personnel can play a protective role against psychological trauma, confirming the importance of peer relationships for resilience and support during and after deployment [5]. Positive interactions at work can enhance connections between work and family, leading to a better work–life balance and preventing conflicts between these two areas [42]. This is particularly important for military personnel engaged in operational missions, who face significant challenges in balancing the achievement of strategic and operational goals with maintaining an adequate quality of life [43].

Research has shown that a feature characterizing good relationships between employees and their superiors is the latter’s propensity to provide growth-oriented feedback [44]. The possibility of receiving feedback on one’s performance is another essential job resource, linked to work-related well-being as it enhances work engagement, which in turn is linked to lower levels of burnout and less need for off-hours recovery [45].

Receiving feedback helps develop a sense of competence and involvement among employees as it values their efforts and contributions [46]. It is a catalyst for sustained engagement, helping individuals recognize their contribution to the organizational mission [47,48]. In addition, feedback plays a key role in clarifying expectations and reducing role ambiguity within military structures, which helps deal with complex scenarios that require quick decision-making and adaptability [49,50]. It is also essential for team dynamics, particularly in military operations where teamwork is critical [44]. Team-level feedback facilitates a shared understanding of individual goals and contributions to collective efforts, enhances collaboration and cohesion, and increases the quality of interpersonal relationships [44]. Regarding relationships with superiors, research has shown that not all feedback produces positive outcomes; rather, its impact depends on contextual factors, including the relationship between supervisors and subordinates and the method of feedback delivery [51,52]. In the armed forces, where hierarchical structures can often inhibit open dialogue, effective upward feedback channels can create pathways of mutual communication that improve trust and commitment among the ranks, improving the overall organizational climate [53]. On an organizational level, promoting an environment where constructive feedback is a well-established practice allows organizations to improve work performance and facilitate professional development, increasing employees’ sense of competence and accomplishment [54]. In the military context, timely and targeted feedback helps personnel better face the challenges of their roles, reducing feelings of inadequacy that could aggravate the conflict between work and personal life [4,54]. Cycles of positive feedback not only boost individual motivation but also promote a culture of openness and continuous improvement, contributing to the development of more effective strategies for managing work demands [55]. Moreover, receiving regular and constructive feedback reinforces the sense of purpose and belonging, essential elements for maintaining morale and ensuring mission-oriented performance [54,55].

A sense of purpose and belonging is an essential element on the path to perceiving one’s work as meaningful [56]. The perception of meaningful work refers to the perception that one’s job is significant and relevant to oneself and others [56,57,58]. Research has shown how perceptions related to work meaningfulness help alleviate the negative effects of fatigue experienced at the end of the workday, probably because when employees perceive their work as meaningful, they are more likely to engage fully and experience job satisfaction [57,58,59]. This awareness can also improve emotional resilience and commitment to one’s role [60,61]. In the context of the armed forces, personnel are often exposed to severe stress and potentially traumatic experiences, which could have a dual effect, either undermining a sense of belonging or increasing it through the realization of the importance of their work to society. Research shows that those who attribute deep meaning to their military role tend to develop greater psychological resilience, which is essential for facing the adversities inherent in military service [62]. The relationship between meaningful work and mental well-being is particularly relevant in the armed forces work environment, where personnel must often deal with traumatic experiences and stressful operational contexts. Studies suggest that meaningful work can act as a buffer against the psychological effects of combat and stress [62]. Additionally, studies show that meaningful work can serve as an intrinsic motivator, causing military personnel to invest more emotionally and psychologically in their tasks, thereby improving performance [63]. Leadership styles in military organizations play a crucial role in promoting perceptions of meaningfulness. For example, an authentic leadership style, characterized by transparency, ethical practices, and emotional intelligence, has been linked to a greater sense of meaningfulness in law enforcement [63]. Viewing one’s leaders as authentic may help personnel improve interpersonal relationships, become more engaged, committed, and satisfied, and ultimately lead to a stronger sense of purpose in their work [64]. In the armed forces, where missions often have profound significance, the sense of duty and purpose can provide members with motivation that enhances job satisfaction and reduces stress, thus decreasing the need for recovery and protecting against significant family conflicts [65]. In addition, the typical circumstances of military life often require personnel to face separation from family and friends, which can create feelings of isolation. In this regard, the perception of meaningful work could act as an anchor, allowing individuals to maintain the connection to personal values and commitments even amid adversity [57,66].

### 1.3. Aims

Over the years, numerous studies have provided insights into the potential psychosocial factors underlying stress in personnel in the armed forces and law enforcement agencies and, more recently, into the protective factors that characterize the profession. However, fewer studies have investigated the emergence of latent profiles, focusing mainly on studying the effects of military life on family dynamics and the emergence of psychopathological risks in spouses and adolescents [67,68]. The interest of the present study is to apply LPA to general working conditions instead.

The research hypotheses are the following:

**H1:**  *Subjects with higher demands and lower resources have higher levels of perceived work–life conflict and need for recovery*.

**H2:**  *Subjects with lower demands and higher resources have lower levels of perceived work–life conflict and need for recovery*.

## 2. Materials and Methods

### 2.1. Sample

This study analyzed a sample of 211 individuals, consisting of 181 men, 23 women, and 7 participants who preferred not to disclose their gender. The sample was collected from various branches of the Italian Armed Forces and law enforcement agencies from April to June 2024. In Italy, the armed forces include the Italian Army, the Navy, the Air Force, and the *Carabinieri*; these are subordinate to the Ministry of Defense and are responsible for the defense of national territory and participation in international missions. Law enforcement, on the other hand, includes the State Police, the *Guardia di Finanza*, the Penitentiary Police, and the Local Police; they depend mainly on the Ministry of the Interior or Justice and are responsible for internal security and the maintenance of public order. The *Carabinieri* represented the largest group, accounting for 49.3% of participants (N = 104), followed by the State Police, which comprised 20.4% (N = 43). Personnel from the Italian Army, including the Air Force and Navy, made up 10.4% of the sample (N = 22), while the remaining 19.9% (N = 42) belonged to other military or law enforcement institutions, such as the *Guardia di Finanza*. The average age was 39.8 years (SD = 12.1), ranging from 20 to 60 years. In terms of education, 55% held a high school diploma, 42.2% had a university degree, and 2.8% had not completed higher education. Regarding work experience, 7.1% of participants had been employed for less than a year, 27.5% had between one and eight years of experience, 6.6% had between eight and fourteen years, and the majority (58.8%) had been working for more than fourteen years. Additionally, participants reported working an average of 41.1 h per week (SD = 8.61) and commuting an average of 22.5 min (SD = 24.9) to reach their workplace.

### 2.2. Measures

For this study, we used the Italian version [69] of the Questionnaire on the Experience and Evaluation of Work 2.0 (QEEW 2.0) [70]. This instrument assesses both job demands and job resources, as well as their negative (strain-related) and positive (motivational) outcomes for employees. The 6-item scale “pace and amount of work” was used to assess the perceived workload (e.g., “I have too much work to do”). Cronbach’s alpha for this study was 0.80. To measure the perception that one’s work is meaningful and relevant to oneself and others, we used the 10-item “meaningful work” scale (e.g., “In my work I can be meaningful to others”). Cronbach’s alpha for this study was 0.85. The 6-item “feedback” scale was used to assess the perception of receiving constructive feedback on the quality of one’s work from supervisors (e.g., “My work provides me with direct feedback on how well I am doing my work”). Cronbach’s alpha for this study was 0.86. Finally, the “relationships with superiors” and “relationships with colleagues” scales (consisting of 6 items each) were used to assess perceptions of having good relationships with superiors and colleagues and satisfaction related to the support received (e.g., “I can count on my supervisors when I encounter difficulties in my work”; “There is a good atmosphere between me and my colleagues”). Cronbach’s alpha values for this study were 0.88 and 0.87, respectively. The 6-item “need for recovery” scale, which measures perceived physical and mental fatigue and difficulty in relaxing at the end of the workday, was used to assess work-related fatigue. An example of an item is “Because of my job, at the end of the working day I feel rather exhausted”. Cronbach’s alpha for this study was 0.85. All items from QEEW 2.0 [69,70] are rated on a 4-point Likert scale with frequency-based anchors for the scales “pace and amount of work,” “feedback,” “relationships with superiors,” “relationships with colleagues,” and “need for recovery” (ranging from 1 = never to 4 = always). The “meaningful work” scale provides anchors based on agreement (ranging from 1 = strongly disagree to 4 = strongly agree).

In addition, the Italian version of the 10-item Work–Family Conflict Scale [71] has been used to measure the spillover effects of work on personal life and personal life on work. An example item is “The demands of my work interfere with my home and family life.” Cronbach’s alpha in this study is = 0.94. Items are rated on a 5-point Likert scale, ranging from 1 = never to 5 = always.

Finally, we used the 10-item Work Safety Scale (WSS) [72], rated on a 5-point Likert scale ranging from 1 = never to 5 = always. This scale was chosen to assess risk perception in one’s work because it adequately captures individuals’ subjective assessments of potential hazards, which were found to be influenced by personal experiences and emotions rather than objective data alone [73]. Cronbach’s alpha in this study was 0.91.

### 2.3. Data Analysis

To test the research hypotheses, a Latent Profile Analysis (LPA) was conducted using RStudio (v4.1.2, R Core Team, Vienna, Austria). LPA is a person-centered statistical method aimed at detecting distinct patterns within multivariate continuous data. In this study, it was chosen to examine the combination of job demand and job resource variables. An iterative modeling process was carried out to test solutions ranging from two to four groups to identify the best-fitting latent structure [74]. Model comparisons were then carried out using the Akaike Information Criterion (AIC), the Bayesian Information Criterion (BIC), and the Sample-size Adjusted Bayesian Information Criterion (SABIC), where lower values indicate a better fit. These indices are used to compare the quality of the models. To verify the classification accuracy, the entropy criterion was used, according to which values as close as possible to 1 indicate a high accuracy [75]. The LPA guidelines indicate that for an appropriate classification, each profile must include at least 30 individuals and must be composed of at least 5% of the sample [76]. Before conducting the LPA, to verify the factorial structure of the measures, a Confirmatory Factor Analysis (CFA) was conducted through RStudio, using the Maximum Likelihood (ML) approach. One-, two-, and six-factor models were tested, respectively, since there were six variables considered in the LPA. As for the fit indices, the Root Mean Square Error of Approximation (RMSEA), the Comparative Fit Index (CFI), the Tucker–Lewis Index (TLI), and the Standardized Root Mean Square Residual (SRMR) were considered. The threshold criteria for assessing the goodness-of-fit are values between 0.05 and 0.08 for RMSEA, values above 0.90 for CFI and TLI, and values below 0.08 for SRMR [77].

Finally, to further verify the final LPA solution and that the differences between the identified profiles were statistically significant, an ANOVA was conducted, followed by post-hoc Tukey and Bonferroni tests to evaluate between-group differences. SPSS (v29, IBM, Armonk, NY, USA) was used to conduct these analyses.

## 3. Results

Table 1 shows the means, standard deviations, and correlations for the research variables. In line with the assumptions and theoretical framework used (JD-R) [10], the correlation matrix shows that job demand variables correlate negatively with job resource variables, and positively with the need for recovery and work–life conflict. Job resource variables, on the other hand, correlate negatively with both outcomes.

To evaluate the structural validity of the variables included in the Latent Profile Analysis (LPA), a Confirmatory Factor Analysis (CFA) was carried out, using the Maximum Likelihood (ML) estimation method. We tested a first model, where all items loaded on one factor, and a two-factor model, where workload and risk perception (job demands) loaded on a first factor, while feedback, meaningful work, relationships with superiors, and relationships with colleagues loaded on a second factor. Finally, in the third model, each item of each variable loaded on its respective factor. The CFA results (see Table 2) showed that the latter six-factor model provided the best fit to the data.

We then proceeded with the Latent Profile Analysis (LPA), testing three models comprising two to four classes. The results suggested that a three-class solution was the best fit to the data. Considering the very low improvement in the AIC and SABIC values of the four-class model, the lower BIC value and the improvement in the entropy value indicate that the three-class model is the best solution (see Table 3).

**Figure 1:**
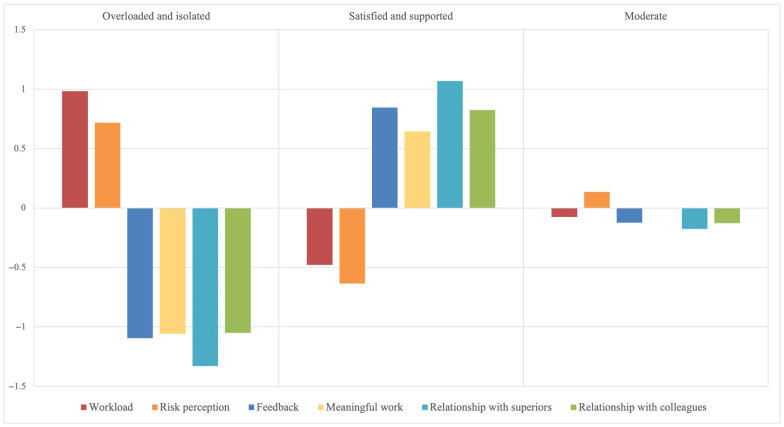
Z-scores for the three profiles.

Figure 1 graphically represents the three profiles.

The first profile, “Overloaded and isolated,” describes individuals who experience a high workload and a high perception of risk associated with their work. At the same time, they receive little feedback from their superiors about their performance, perceive their work as not very meaningful, and have weak relationships with both superiors and colleagues. This combination suggests a situation of high stress, low job satisfaction, and poor social support in the workplace. This profile represents 20% of the sample (N = 41). The second profile, “Satisfied and supported,” describes individuals who report a low workload and low perception of risks associated with their work. They report receiving constructive feedback from superiors, find their work meaningful, and have good relationships with both superiors and coworkers. This profile indicates a healthy work environment with high satisfaction and strong social support, while potentially stressful variables are at low levels. This profile represents 32% of the sample (N = 68). The third profile, “Moderate,” describes individuals with average levels in all variables considered: workload, risk perception, feedback, meaningful work, and interpersonal relationships with superiors and colleagues. This suggests a balanced condition, without markedly positive or negative extremes, and may indicate a stable but potentially improvable work situation. Although the workload is low, the perception of risks associated with work is slightly higher, which could be a warning sign, especially as it is associated with low levels of job resources. This profile represents 48% of the sample (N = 102). The first profile shows higher mean scores in job demands (workload: M = 2.63, SD = 0.41; risk perception: M = 3.85, SD = 0.71) and lower mean scores in job resources (feedback: M = 1.89, SD = 0.38; meaningful work: M = 2.78, SD = 0.39; relationship with superiors: M = 2.28, SD = 0.33; relationship with colleagues: M = 2.72, SD = 0.53). Therefore, in line with the JD-R model [10], we would expect individuals belonging to this class to show higher levels of need for recovery and work–family conflict. The second profile, on the contrary, shows higher mean scores in job resources (feedback: M = 3.20, SD = 0.51; meaningful work: M = 3.53, SD = 0.34; relationship with superiors: M = 3.74, SD = 0.28; relationship with colleagues: M = 3.70, SD = 0.35) and lower mean scores in job demands (workload: M = 1.88, SD = 0.47; risk perception: M = 2.65, SD = 0.81). Therefore, we would expect lower levels of need for recovery and work–family conflict for workers belonging to profile two. Finally, the third profile shows mean scores that lie between the scores of the first profile and those of the second profile across all variables (workload: M = 2.09, SD = 0.41; risk perception: M = 3.34, SD = 0.78; feedback: M = 2.54, SD = 0.52; meaningful work: M = 3.24, SD = 0.36; relationship with superiors: M = 2.98, SD = 0.33; relationship with colleagues: M = 3.20, SD = 0.34). In this case, we would expect average levels of need for recovery and work–family conflict. To test these hypotheses and the statistical significance of the differences between the means, an ANOVA was conducted. The ANOVA results show that the means of both dependent variables considered differ statistically significantly among the three profiles (need for recovery: F(2,208) = 26.448, *p*-value < 0.001; work–family conflict: F(2,208) = 32.677, *p*-value < 0.001). Tukey’s and Bonferroni’s post-hoc tests confirmed the statistical significance of the differences between the means of the three groups for the variables considered. The consistency between the results of the two multiple comparison tests reinforces the validity of the observed differences, suggesting that the classification obtained from the LPA is statistically robust. The means of the dependent variables for the three profiles are reported in Table 4.

As hypothesized, the “Overloaded and isolated” profile shows significantly higher means of need for recovery and work–family conflict compared to the other groups, confirming the H1 hypothesis. The “Satisfied and supported” profile shows the lowest mean scores in both variables, confirming the H2 hypothesis. As also hypothesized, the “Moderate” profile shows levels of need for recovery and work–family conflict halfway between profile one and profile two.

## 4. Discussion

The present study aimed to apply the Job Demands–Resources (JD-R) model [10] to personnel of the armed forces and law enforcement agencies, exploring different profiles based on perceived demands and resources. The JD-R model suggests that an imbalance between job demands and resources may contribute to triggering health deterioration processes, which are associated with negative work outcomes [11,12,13,14,15]. Based on this, we hypothesized that individuals facing higher job demands and fewer resources might experience greater work–life conflict and a stronger need for recovery, while those with lower demands and greater resources might experience the opposite. We therefore sought to identify demands and resources that could best capture the specifics of the police and armed forces’ work. Based on the existing literature, the workload has been found to be particularly stressful, as it directly contributes to the depletion of physical and mental resources [16,23], increases fatigue [15,17], and negatively affects personal life [12,16]. Another stressor specific to the armed forces is work-related risks. Perceiving one’s job as risky creates states of anxiety and agitation [28,29] and stands as a risk to physical and mental health, with spillover effects on family life as well [24,25,26]. Workload and perceived work-related risks have therefore been identified as job demand variables.

Regarding resources, the literature has shown that greater personal and work resources are correlated with lower health risks and better off-work living conditions [36]. In the armed forces context, finding support among colleagues fosters camaraderie and the sharing of work-related risks and anxieties, and improves mental health through better coping strategies and resilience [4,39,40]. Having good relationships with superiors is equally crucial in the armed forces environment, which is characterized by rigid hierarchies and rules [41]. Closely related to this, the literature has shown that superiors who give constructive feedback create more engaged and effective workers, thus improving communication and climate [53]. Perceiving a healthy and positive climate, in turn, positively affects the perceived meaningfulness of one’s work [60,61], which has a protective effect on work-related stress [62]. The work of the armed forces and law enforcement agencies plays a positive role in society and is related to a strong sense of duty stemming from having as its goal the protection and security of citizens [65]. The literature has shown that the awareness of performing a profession relevant to oneself and others has a protective role, even in the occurrence of adverse events [57,66]. For these reasons, relationships with colleagues and superiors, the presence of feedback, and the meaningfulness of work were identified in the present study as job resource variables.

As dependent variables, we identified the need for recovery and work–life conflict as indicators of stress. The need for recovery is a concept intrinsically linked to the fatigue experienced during the workday [6]. Work activities that wear down workers’ physical and mental resources generate fatigue that, if not adequately recovered at the end of the workday and if accumulated over time, can lead to severe health problems [6,28,30]. Since recovery should take place during free time, excessive levels of fatigue may prevent detachment from work-related thoughts and the enjoyment of quality leisure time [6]. In such cases, difficulty relaxing could negatively affect family and personal life, increasing conflict and spillover effects between the spheres of work and private life [12].

To explore the distribution of the examined indicators and test the research hypotheses, a Latent Profile Analysis (LPA) was conducted on the demand and resource variables. Subsequently, mean comparison analyses were performed to verify the levels of the dependent variables based on group membership that emerged from the LPA. The LPA identified three distinct profiles among armed forces personnel, and the ANOVA and post-hoc tests confirmed that the differences in levels of need for recovery and work–family conflict among these profiles were statistically significant. The initial research hypotheses were both confirmed. Specifically, the first profile, “Overloaded and isolated,” characterized by high job demands and low job resources, showed higher levels of work–family conflict and need for recovery. The second profile, “Satisfied and supported,” characterized by high job resources and low job demands, showed lower levels of need for recovery and work–family conflict. Finally, the third profile, “Moderate,” with moderate demands and resources, showed an intermediate level of need for recovery and work–family conflict, indicating a balanced but potentially vulnerable work situation. The profiles proved to be in line with the theorization of the JD-R model [10]. Perceiving high workloads and that one’s work is linked to various risks, in the absence of work resources such as receiving feedback and support from one’s superiors and colleagues, and personal resources such as the perception of doing meaningful work, can lead to greater fatigue at the end of the workday and worsened work–family conflict. Both outcomes are closely linked, as feelings of fatigue can affect the quality and quantity of time spent on other occupations or in the family sphere [6]. Work–life conflict as a result of particularly stressful moments belonging to one or the other area can also, in turn, accentuate the fatigue experienced during the workday, consequently increasing the need to recover physical and mental energy [6]. This interaction reinforces the necessity for organizations to prioritize the provision of adequate resources to employees. When work and personal resources are present, especially in combination with low workloads or perceptions of manageable work-related risks, work–life conflict and levels of need for recovery may also improve [11]. From a practical point of view, these findings support the hypothesis that improving well-being at work requires not only a reduction in demands—which is very often difficult if not impossible to implement—but also the identification and increase of available resources. Introducing effective feedback mechanisms, improving communication to avoid hierarchical barriers, and promoting a culture that emphasizes meaningful work and support among colleagues can act collectively to improve employee well-being. Addressing these issues through targeted mental health strategies and supportive organizational structures can help mitigate the interconnected issues of perceived risk and work-related stress. In addition, addressing the challenges of work–life conflict through supportive management practices that foster flexible work formulas can enable military personnel to better integrate their work and personal lives, promoting a sense of security and commitment. These suggestions are in line with the literature, which has indicated that acting on workload management [12,16], increased social support [37,38], receipt of constructive feedback [44], and a stronger sense of meaning [56,57,58] can help to effectively manage stress and increase employees’ sense of competence and accomplishment [48,54].

### Limitations and Directions for Future Research

Several limitations should be acknowledged. First, the sample size, while adequate for exploratory Latent Profile Analysis (LPA), may limit the generalizability of the identified profiles. Although recent methodological literature suggests that LPA can be conducted with smaller samples under certain conditions, such as few and well-differentiated profiles [78], a larger sample would strengthen the robustness of the results and allow for more nuanced differentiation of profiles. Second, the sample consists exclusively of Italian Armed Forces personnel, which limits the external validity of the results. Cultural, institutional, and operational factors specific to the Italian population may influence the structure and interpretation of the latent profiles. Third, the cross-sectional design precludes any inference about the temporal stability of the profiles or the directionality of associations between profile membership and outcome variables. Finally, future research should consider including a broader range of psychological, behavioral, and contextual variables to gain a more comprehensive understanding of individual differences within armed forces populations.

## 5. Conclusions

There is a large body of literature on the role of excessive job demands in generating work-related stress among security workers [79]. The relevance of the JD-R model [10,11] in armed forces contexts is underscored by its capacity to design targeted interventions aimed at improving the psychological well-being of these personnel [80]. By systematically identifying job demands and enhancing available resources, armed forces organizations can create healthier work environments that prioritize mental health along with operational effectiveness. This research aims to emphasize the importance of adequate personal (e.g., the perceived importance of one’s job role) and organizational resources (e.g., supportive leadership or social camaraderie). Since security workers cannot be relieved of some forms of demands that are inherently part of their daily duties, the attention to these resources can mitigate the adverse effects expected to occur in such emotionally demanding settings and promote resilience among service members. High-stress environments, combined with the unique pressures of military operations, require a thorough understanding of how these demands interact with available resources. In response to this need, tailored interventions are increasingly recognized as essential to foster a supportive environment that improves the overall well-being of service members [13]. Research has identified several personal resources, such as personal resilience [81], optimism, and self-regulation [82], that have been shown to moderate the relationship between job demands and employee well-being in the military context. Our contribution aims to highlight the importance of meaningfulness of work as a central aspect in the balance between excessive demands and a possible lack of resources on psychological well-being. Since meaningfulness is an individual perception and not a personality factor, a practical implication may be to focus on experiential training activities that highlight the social value of safety-related professions through initiatives aimed at bringing out shared values. This would also strengthen teamwork and a culture of feedback and interpersonal support. By implementing these interventions, the armed forces could cultivate a more supportive and responsive environment for the mental health care of their personnel. It is also useful to draw attention to the importance of personnel selection policies that adequately highlight the need to share the intrinsic values of these professions. Balancing the focus on practical aspects with emotional and relational ones, and focusing on an in-depth investigation of shared values, could be useful both in terms of results for armed forces organizations and in avoiding exposing workers to pressures they are not comfortable with.

## Figures and Tables

**Table 1:** Means, standard deviations, and correlations between the study variables.

Variable	Mean (SD)	1	2	3	4	5	6	7	8
1. Workload	2.13 (0.52)	1							
2. Risk perception	3.18 (0.87)	0.376 **	1						
3. Meaningful work	3.24 (0.44)	−0.273 **	−0.167 *	1					
4. Feedback	2.63 (0.67)	−0.267 **	−0.336 **	0.532 **	1				
5. Relationship with colleagues	3.27 (0.52)	−0.340 **	−0.312 **	0.352 **	0.412 **	1			
6. Relationship with superiors	3.09 (0.61)	−0.455 **	−0.426 **	0.508 **	0.562 **	0.599 **	1		
7. Need for recovery	1.85 (0.53)	0.638 **	0.436 **	−0.278 **	−0.230 **	−0.327 **	−0.427 **	1	
8. Work–life conflict	2.30 (0.62)	0.695 **	0.538 **	−0.304 **	−0.292 **	−0.354 **	−0.507 **	0.743 **	1

Notes: ** Correlation is significant at the 0.01 level; * correlation is significant at the 0.05 level (2-tailed).

**Table 2:** Indices of fit of the Confirmatory Factor Analysis.

Models	Model Fit					
	χ^2^	df	CFI	TLI	RMSEA (90% CI)	SRMR
1-factor model	3440.772	860	0.473	0.447	0.119 (0.115–0.123)	0.122
2-factor model	2858.950	858	0.592	0.571	0.105 (0.101–0.109)	0.116
6-factor model	1254.602	832	0.915	0.906	0.049 (0.043–0.055)	0.078

**Table 3:** Statistics for the latent profile structures.

Models	AIC	BIC	SABIC	Entropy
2-class model	2021.260	2105.975	2028.741	0.79
3-class model	1909.701	2036.849	1916.465	0.85
4-class model	1906.305	2077.917	1915.352	0.84

**Table 4:** Means and standard deviations of the dependent variables for the three latent profiles.

Profiles	Mean (SD)	
	Need for Recovery	Work–Family Conflict
Profile 1 “Overloaded and isolated”	2.25 (0.59)	2.83 (0.55)
Profile 2 “Satisfied and supported”	1.58 (0.44)	1.95 (0.62)
Profile 3 “Moderate”	1.86 (0.44)	2.31 (0.50)

Note: All differences between means are statistically significant at *p* < 0.001 according to Tukey and Bonferroni post-hoc tests.

## Data Availability

The data presented in this study are available upon request from the corresponding author due to privacy reasons.
